# Knowledge and Attitudes of Parkinson's Disease in Rural and Urban Mukono District, Uganda: A Cross-Sectional, Community-Based Study

**DOI:** 10.1155/2015/196150

**Published:** 2015-11-25

**Authors:** Mark Kaddumukasa, Angelina Kakooza, Martin N. Kaddumukasa, Edward Ddumba, Levi Mugenyi, Martha Sajatovic, Elly Katabira

**Affiliations:** ^1^Department of Medicine, School of Medicine, Makerere University College of Health Sciences, P.O. Box 7072, Kampala, Uganda; ^2^Department of Paediatrics, School of Medicine, Makerere University College of Health Sciences, P.O. Box 7072, Kampala, Uganda; ^3^Department of Medicine, St. Francis Nsambya Hospital, Nkozi University, P.O. Box 7146, Kampala, Uganda; ^4^Infectious Diseases Research Collaboration, Mulago Hill Road, MUJHU3 Building, P.O. Box 7475, Kampala, Uganda; ^5^University Hospitals Case Medical Centre, Neurological Institute, Case Western Reserve University, 11100 Euclid Avenue, Cleveland, OH 44106, USA; ^6^Neurological and Behavioural Outcomes Centre, University Hospitals Case Medical Centre, 11100 Euclid Avenue, Cleveland, OH 44106, USA

## Abstract

*Background*. Parkinson's disease (PD) negatively affects the quality of life. There is limited information on PD published from Africa. Lack of adequate knowledge poses a barrier in the provision of appropriate treatment and care for individuals with PD.* Methods*. A cross-sectional survey was conducted in urban and rural Mukono district, central Uganda. Through the systematic sampling method, data were gathered from 377 adult participants, interviewed on selected aspects of PD knowledge and attitudes.* Results*. Of the 377 participants, 47% were from urban settings and 68% (260/377) were women with a median age (IQR) of 34 (26–48) years. Half of the study respondents did not know the body part involved in or apparent cause of PD. Nearly 1/3 of individuals believed that PD is a form of insanity and 17% believed that PD is contagious. Rural dwellers were more likely to have incorrect knowledge regarding selected aspects of PD.* Conclusions*. Understanding the cause of PD is very limited in our setting. Some beliefs about PD aetiology may potentially worsen stigma and social isolation. This study highlights the need for increasing PD awareness in our settings. Public health approaches that improve knowledge are urgently needed to promote care access and community response to Parkinson's disease.

## 1. Introduction

Few studies regarding Parkinson's disease (PD) have been performed in Sub-Saharan Africa, [1–4] and no studies have evaluated community knowledge and attitudes to PD in our setting. Reportedly, there is a low prevalence of PD within black African populations compared to European and North American populations. This might be reflecting a difference in the susceptibility in this population or lack of information in this population [4–6]. Alternatively, prevalence studies might be limited due to underreporting of PD and PD symptoms among individuals who fear societal stigma related to having PD. Studies have described PD stigma and its impact on the social activities of the sufferers. Subjects living in rural areas were more stigmatized than urban dwellers in these studies [7, 8]. Despite advancements in treatment, diagnosis, and care of Parkinson's disease patients', lack of adequate knowledge and associated beliefs and attitudes among the community dwellers might have an important role in limiting access to proper treatment and care. Understanding community knowledge and beliefs in a rural and urban setting in central Uganda could help to inform care approaches that reduce stigma and promote care seeking. We therefore set out the main objective of this study, to assess knowledge and attitudes towards PD in a Ugandan community sample.

## 2. Methods and Materials

### 2.1. Study Site

The study was conducted within urban and rural Mukono, with Mukono town council as urban and Kojja subcounty as rural.

### 2.2. Study Design

This was a cross-sectional study conducted within an ongoing population survey of 3000 participants on prevalence and incidence of neurological diseases in Mukono district. This study was part of a larger US National Institute of Heath-Funded Medical Education Partnership Initiative (MEPI) neurological disorder survey, where we assessed community knowledge and attitudes on Parkinson's disease among some of the study participants.

### 2.3. Patient Recruitment

Face to face interviews were conducted between August and November 2014. Multistage stratified sampling technique was used as described below. At the subcounty level, urban Mukono Town Council was randomly selected and Kojja subcounty (rural) in Mukono district. Mapping of the selected urban and rural areas was based on the Uganda population and Housing Census where 11,373 and 13930 households were identified in Mukono TC and Ntenjeru, respectively. The sampling frame was all households in these areas. Systematic sampling was used to select households in each village to total 2000 in the urban area and 2200 in the rural area that would participate in the large population survey. One adult, randomly selected from each household, was approached and consented and participated in the study. The selected households were visited by the research team. The randomly selected participant was informed about the research and invited to participate in the survey.

### 2.4. Procedures

A request for a written informed consent was sought from all potential participants. Consenting participants were taken through a pretested screening questionnaire and those having symptoms suggesting neurological diseases were asked to come for follow-up check at a local health facility the following morning from where they were further evaluated by a team of neurologists. Blood pressure and anthropometric measurements were taken for each participant. Out of the 1500 participants in the urban area and 1500 in the rural area, systematic sampling was used to select 177 and 200 participants from the urban and rural areas, respectively, to be interviewed on selected aspects of PD knowledge and attitudes. The study was conducted from August 2014 to December 2014. The inclusion criteria included usual resident who is present in the sampled household on the night before the survey, being aged 18 years or older (adult), and willingness to provide informed consent. We excluded those who were physically unable to undergo interview. One adult, randomly selected from each household, was approached and consented and participated in the study. Consenting participants were taken through a pretested screening questionnaire. To address potential sources of bias, a standardized questionnaire was used with a wide range of responses for the study participants which were read to the participants. The interviewers were trained using standardized protocols for data collection to minimize interobserver variability during data gathering and entering data.

### 2.5. Sample Size Determination

The sample size was calculated using the following formula: {*n* = *Z*_*α*_^2^(*pq*)/*d* ^2^} where *p* is prevalence of hypertension, *q* is complement of the prevalence, margin of error is error which is *d*, and alpha is significance level. Setting significance at 0.05 and error margin at 5%, adjusted sample requirement for an assumed 10% level of nonresponse (nr = 10%) = *N* ^*∗*^. Based on a previous study in Mukono [9] where hypertension prevalence was 27% and *N* ^*∗*^ = 336, we recruited 370 participants.

### 2.6. Ethical Considerations

Ethical approval for the study was obtained from Makerere University College of Health Sciences' School of Medicine review board and ethics committee reference number 2013-145 and Uganda National Council of Science and Technology reference number HS1551. Written informed consent was obtained before enrolling the participants into the study.

### 2.7. Questionnaire

The questions were divided into two parts covering PD aetiology and PD symptomatology. The PD-focused questionnaire consisted of 9 questions with various options that were read out to the participants. The questions that were asked included one question on body part affected in PD, one question on the cause of PD, four questions on symptomatology of PD, one question on beliefs in PD, one question on attitude on work in PD, and one question on mode of transmission in PD. Data regarding name, age, and educational status was also collected. Most questions were simple open-ended questions requiring “yes” or “no” responses (Supplementary Material: Study Questionnaire, in Supplementary Material available online at http://dx.doi.org/10.1155/2015/196150).

### 2.8. Data Analysis

Descriptive statistics of frequencies and percentages (in addition to median for age) were used to summarize data on sociodemographic variables and Parkinson's disease knowledge and practice. Those with correct responses were compared to those with wrong answers to determine any associated factors using chi-square test. All tests were two tailed with a level of significance at 0.05. All statistical analyses were performed using STATA software version 12 (Stata Corporation, College Station, TX, USA).

## 3. Results

As illustrated in Table 1, 377 adults were enrolled with 177 (47%) from the urban settings. Sixty-eight percent (260/377) were women with a median age (IQR) of 34 (26–48) years.

### 3.1. Knowledge on Parkinson's Disease

As illustrated in Table 2, 50.4% (190/377) of participants did not know the body part related to PD. Nearly nineteen percent (71/377) reported that degeneration was responsible for the disease, while 10.1% (38/377) reported it was due to lack of blood supply to the brain. Four percent of the participants reported that it was due to infections. The majority of study participants (273/377) were able to report the main symptom of Parkinson's disease as tremors and nearly 61% (228/377) reported that the body becomes stiff. Only 15.1% of the study participants (57/377) could not identify symptoms of PD. About half of the respondents (191/377) reported that depression is common among those with PD.

### 3.2. Attitudes Regarding Parkinson's Disease

A majority of community dwellers (239/377, 63%) were able to recognise that PD is not contagious. However, 17% (64/377) reported that PD is and 28.7% believed that PD is a form of insanity. About 39% believed that someone with PD can still work while 46.7% did not feel that individuals with PD could work.

### 3.3. Associations between Participants' Response and Demographics

Correct belief regarding the brain being the main body part involved was significantly higher among respondents aged less than 35 years and urban residency with *p* = 0.04 and 0.021, respectively. Urban residents significantly reported that the body becomes stiff or stiffness and tremor were part of the symptoms of PD compared to their rural counterparts with *p* < 0.001 and *p* = 0.003, respectively. There were no associated differences in responses to main symptoms of PD, associations with depression and insanity, and whether PD was contagious (Tables 3(a) and 3(b)).

## 4. Discussion

This first report on community knowledge and attitudes towards PD in Uganda suggests that while most individuals recognize typical symptoms of PD, the awareness of causes of PD remains limited. Minority have beliefs about PD that may worsen stigma and social isolation. Without accurate understanding of the basic etiology of PD and the comprehensive scope of its impact in our settings, community responses to this neurodegenerative disease often are influenced by misconceptions based on myths and false assumptions. For example, it is believed in some communities that PD is associated with touching the mother-in-law.

From this study, it is evident that PD awareness is low in central Uganda. Half of the study respondents were not knowledgeable regarding the body part involved in PD. The majority of respondents were, however, able to identify tremors as a major symptom in PD. This is different from a study conducted in Tanzania, where majority of the study participants were not aware of the symptoms and appropriate treatment for PD [2]. This might be due to population differences or effects of previous efforts by various health educational groups which have increased health awareness especially addressing issues related to misconceptions regarding PD. However, in this study rural dwellers were more likely to have incorrect knowledge about PD. Most people do not consider themselves likely to be susceptible to PD [10].

A key finding from this survey was that 17% of participants believed that PD was contagious while 28% believed that PD is a form of insanity. It might be expected that these attitudes contribute to stigma associated with PD. This is similar to earlier work which reported that half of the respondents saw stigma attached to this disease [10]. These stigmatizing misconceptions make it likely that individuals with PD might conceal their symptoms and delay care seeking, making their situation even more challenging [11]. Designing awareness campaigns for increasing knowledge and changing attitudes within communities would help in increasing social support for PD sufferers in our settings. This study emphasizes the need for adequate education and imparting accurate knowledge to communities. In an earlier study, participants with stigma were more likely to have negative attitudes to people with PD. Stigma leads to discrimination which may be associated with depression and poorer adjustment among those with the stigmatized condition. We did not find any significant differences between rural and urban communities regarding stigma. However, some studies have reported that stigma was higher in rural areas than urban areas [10].

Nearly half of survey respondents did not feel that those with PD could work for a living. The physical, psychological, economic, and social suffering caused by PD makes individuals dependent on their families for support [12, 13]. Since PD seems to be stigmatized in the community, it is possible that some individuals might make efforts to conceal their PD from others, at least in the early stages when it is less noticeable. Perhaps, at least part of the reason why PD is so widely believed to be disabling by the community is that PD that is recognized tends to be later-stage illness.

## 5. Conclusion

PD, at least in its more severe form, is readily recognized by the community in Uganda; however, knowledge about what causes PD is limited and stigmatizing misconceptions are unfortunately relatively common. Awareness and education programs on PD have an important role in improving accurate understanding of illness and in reducing stigma. This may ultimately affect care seeking and health outcomes among those with PD. Further studies are needed to understand how knowledge and attitudes may influence care seeking and access for individuals with PD in Sub-Saharan Africa.

## Supplementary Material

Supplementary material showing the study questionnaire used in this study. The questionnaire had two parts covering PD aetiology and PD symptomatology. The PD-focused questionnaire consisted of 9 questions, including the body part affected, symptoms and mode of transmission.

## Figures and Tables

**Table 1:** Demographic characteristics of the study participants (*N* = 377).

Characteristic	
Median age in years (IQR)	34 (26–48)
Age range in years (min, max)	18–85
Age categories in years, *n* (%)	
<25	74 (19.6)
25–34	125 (33.2)
35–44	66 (17.5)
>44	112 (29.7)
Gender, *n* (%)	
Male	123 (32.6)
Residence, *n* (%)	
Rural	200 (53.1)

**Table 2:** Knowledge of Parkinson's disease (*N* = 377).

	*N* (%)
Part of the body related to Parkinson's disease	
Liver	1 (0.3)
Kidney	6 (1.6)
Brain	141 (37.4)
Heart	39 (10.3)
Do not know	190 (50.4)
Parkinson's disease is due to	
Degeneration	71 (18.8)
Lack of blood supply	38 (10.1)
Swelling	66 (17.5)
Infection	15 (4.0)
Do not know	187 (49.6)
Main symptom of Parkinson's disease	
Reduced eye sight	11 (2.9)
Loss of memory	20 (5.3)
Tremors in the hands	273 (72.4)
Unsteadiness of the legs	16 (4.2)
Do not know	57 (15.1)
Correct statement about Parkinson's disease	
Common in children	6 (1.6)
Body becomes stiff	228 (60.5)
Spreads from person to another	3 (0.8)
More common in women	52 (13.8)
Do not know	88 (23.3)
Is depression common among those with Parkinson's disease?	
Yes	191 (50.7)
No	110 (29.2)
Do not know	76 (20.2)
Tremor and muscle stiffness are main symptoms of Parkinson's disease	
Yes	266 (70.6)
No	38 (10.1)
Do not know	73 (19.4)
Parkinson's disease is a form of insanity	
Yes	108 (28.7)
No	159 (42.2)
Do not know	110 (29.2)
Parkinson's disease is contagious	
Yes	64 (17.0)
No	239 (63.4)
Do not know	74 (19.6)
Cannot work if you have Parkinson's disease	
Yes	147 (39.0)
No	176 (46.7)
Do not know	54 (14.3)

**(a):** 

Study question	Correct answer *N* (%)	Wrong answer or do not know *N* (%)	*P* value
Part of the body related to Parkinson's disease (brain)			
Age categories in years			
<35	84 (42.2)	115 (57.8)	**0.041**
≥35	57 (32.0)	121 (68.0)	
Gender			
Male	50 (40.7)	73 (59.3)	0.364
Female	91 (35.8)	163 (64.2)	
Area of residence			
Rural	64 (32.0)	136 (68.0)	**0.021**
Urban	77 (43.5)	100 (56.5)	
Parkinson's disease is due to (degeneration)			
Age categories in years			
<35	37 (18.6)	162 (81.4)	0.900
≥35	34 (19.1)	144 (80.9)	
Gender			
Male	21 (17.1)	102 (82.9)	0.543
Female	50 (19.7)	204 (80.3)	
Area of residence			
Rural	36 (18.0)	164 (82.0)	0.660
Urban	35 (19.8)	142 (80.2)	
Main symptom of Parkinson's disease (tremors in the hands)			
Age categories in years			
<35	147 (73.8)	52 (26.1)	0.504
≥35	126 (70.8)	52 (29.2)	
Gender			
Male	89 (72.4)	34 (27.6)	0.986
Female	184 (72.4)	70 (27.6)	
Area of residence			
Rural	140 (70.0)	60 (30.0)	0.265
Urban	133 (75.1)	44 (24.9)	
Correct statement about Parkinson's disease (body becomes stiff)			
Age categories in years			
<35	127 (63.8)	72 (36.2)	0.161
≥35	101 (56.7)	77 (43.3)	
Gender			
Male	80 (65.0)	43 (35.0)	0.207
Female	148 (58.3)	106 (41.7)	
Area of residence			
Rural	96 (48.0)	104 (52.0)	**<0.001**
Urban	132 (74.6)	45 (25.4)	
Is depression common among those with Parkinson's disease? (yes)			
Age categories in years			
<35	105 (52.8)	94 (47.2)	0.388
≥35	86 (48.3)	92 (51.7)	
Gender			
Male	66 (53.7)	57 (46.3)	0.418
Female	125 (49.2)	129 (50.8)	
Area of residence			
Rural	93 (46.5)	107 (53.5)	0.086
Urban	98 (55.4)	79 (44.6)	

**(b):** 

Study question	Correct answer *N* (%)	Wrong answer or do not know *N* (%)	*P* value
Tremor and muscle stiffness are main symptoms of Parkinson's disease (true)			
Age categories in years			
<35	138 (69.4)	61 (30.6)	0.586
≥35	128 (71.9)	50 (28.1)	
Gender			
Male	88 (71.5)	35 (28.5)	0.770
Female	178 (70.1)	76 (29.9)	
Area of residence			
Rural	128 (64.0)	72 (36.0)	**0.003**
Urban	138 (78.0)	39 (22.0)	
Is Parkinson's disease a form of insanity? (no)			
Age categories in years			
<35	84 (42.2)	115 (57.8)	0.988
≥35	75 (42.1)	103 (57.9)	
Gender			
Male	54 (43.9)	69 (56.1)	0.636
Female	105 (41.3)	149 (58.7)	
Area of residence			
Rural	91 (45.5)	109 (54.5)	0.165
Urban	68 (38.4)	109 (61.6)	
Is Parkinson's disease contagious? (no)			
Age categories in years			
<35	119 (59.8)	80 (40.2)	0.125
≥35	120 (67.4)	58 (32.6)	
Gender			
Male	73 (59.4)	50 (40.6)	0.256
Female	166 (65.4)	88 (34.6)	
Area of residence			
Rural	134 (67.0)	66 (33.0)	0.122
Urban	105 (59.3)	72 (40.7)	
Can someone work if they have Parkinson's disease? (yes)			
Age categories in years			
<35	72 (36.2)	127 (63.8)	0.237
≥35	75 (42.1)	103 (57.9)	
Gender			
Male	48 (39.0)	75 (61.0)	0.993
Female	99 (39.0)	155 (61.0)	
Area of residence			
Rural	85 (42.5)	115 (57.5)	0.138
Urban	62 (35.0)	115 (65.0)	

## Strengths and Limitations

We realize that our study had a number of limitations including cross-sectional design, relatively small sample size, and reliance on standardized and closed-ended assessments. Our study could have benefited from longitudinal ethnographic data exploring further the experiences and meaning of PD to the general life of sufferers and their care providers within the community context. However, the studied population is representative of the general population, including the rural and urban settings in Uganda, and findings provide a reasonable stepping stone for further research.
