# Supplementary material for: Knowledge and Attitudes of Parkinson's Disease in Rural and Urban Mukono District, Uganda: A Cross-Sectional, Community-Based Study
Source: Parkinsons Dis. 2015 Nov 25;2015:196150. doi: 10.1155/2015/196150 (PMC4673351; doi:10.1155/2015/196150)
Supplement: Supplementary file 1 — Supplementary material showing the study questionnaire used in this study. The questionnaire had two parts covering PD aetiology and PD symptomatology. The PD-focused questionnaire consisted of 9 questions, including the body part affected, symptoms and mode of transmission. [file 196150.f1.docx]

Parkinson’s disease

| **Study question** | Read all responses to the study participant |
| --- | --- |
| 1. **What part of the body is related to Parkinson’s disease** | 1. Liver 2. Kidney 3. Brain 4. Heart 5. Don’t know |
| 1. **Parkinson’s disease is due to** | 1. Degeneration 2. Lack of blood supply 3. Swelling 4. Infection 5. Don’t know |
| 1. **The main symptom of Parkinson’s disease is?** | 1. Reduced eye sight 2. Loss of memory 3. Tremors in the hands 4. Unsteadiness of the legs 5. Do not know |
| 1. **What is the correct statement about Parkinson's disease?** | 1. Common in children 2. Body becomes stiff 3. Spreads from person to another 4. More common in women 5. Do not know |
| 1. **Is depression common among those with Parkinson’s disease?** | 1. Yes 2. No 3. Do not know |
| 1. **Tremor and muscle stiffness are main symptoms of Parkinson’s disease** | 1. Yes 2. No 3. Do not know |
| 1. **Parkinson's disease is a form of insanity** | 1. Yes 2. No 3. Don’t know |
| 1. **Is Parkinson's disease contagious?** | 1. Yes 2. No 3. Don’t know |
| 1. **Can you work if you have Parkinson's disease?** | 1. Yes 2. No 3. Do not know |
